# Isochoric supercooled preservation and revival of human cardiac microtissues

**DOI:** 10.1038/s42003-021-02650-9

**Published:** 2021-09-22

**Authors:** Matthew J. Powell-Palm, Verena Charwat, Berenice Charrez, Brian Siemons, Kevin E. Healy, Boris Rubinsky

**Affiliations:** 1 Department of Mechanical Engineering, University of California at Berkeley, Berkeley, CA 94720 USA; 2 Department of Bioengineering, University of California at Berkeley, Berkeley, CA 94720 USA; 3 Department of Materials Science and Engineering, University of California at Berkeley, Berkeley, CA 94720 USA

**Keywords:** Tissue engineering, Lab-on-a-chip

## Abstract

Low-temperature biopreservation and 3D tissue engineering present two differing routes towards eventual on-demand access to transplantable biologics, but recent advances in both fields present critical new opportunities for crossover between them. In this work, we demonstrate sub-zero centigrade preservation and revival of autonomously beating three-dimensional human induced pluripotent stem cell (hiPSC)-derived cardiac microtissues via isochoric supercooling, without the use of chemical cryoprotectants. We show that these tissues can cease autonomous beating during preservation and resume it after warming, that the supercooling process does not affect sarcomere structural integrity, and that the tissues maintain responsiveness to drug exposure following revival. Our work suggests both that functional three dimensional (3D) engineered tissues may provide an excellent high-content, low-risk testbed to study complex tissue biopreservation in a genetically human context, and that isochoric supercooling may provide a robust method for preserving and reviving engineered tissues themselves.

## Abstract (web summary)

Powell-Palm et al. demonstrate sub-zero centigrade preservation and revival of autonomously beating, 3D human induced pluripotent stem cell (hiPSC)-derived cardiac microtissues via isochoric supercooling, without the use of chemical cryoprotectants. Their study suggests that functional 3D engineered tissues may provide a high-content, low-risk testbed to study complex tissue biopreservation in a genetically human context, and that isochoric supercooling may provide a robust method for preserving and reviving engineered tissues themselves.

## Introduction

Modern organ and tissue transplantation efforts in the U.S. and abroad are hamstrung by the unavailability of on-demand donor biologics^1–3^. While this may be interpreted in part as a matter of policy, the central scientific driver of this organ and tissue deficit is the extremely short ex vivo shelf life of complex biologics. In the U.S. alone, though more than 100,000 people are currently on transplant waiting lists, more than 70% of all thoracic donor organs are discarded each year, simply due to an inability to preserve them for sufficient time periods^4^. The preservable ex vivo lifetime of donor hearts for instance, which are typically held on ice in “static cold storage”, is only 4–6 h^1^.

While historical research on the preservation of organs and complex tissues focused largely on the deep cryogenic temperatures that would theoretically enable indefinite storage, the last decade has seen the advent of “high-subzero” preservation efforts, targeting the −20°–0 °C range^5^. These efforts aim simply for multiday preservation, in recognition of the fact that even 24- to 48-h preservation of many complex donor biologics could prove transformative for transplant medicine^1–4^. Chief amongst high-subzero efforts stands supercooled storage, which aims, via a number of different protocols, to hold biologics in an ice-free, metastable liquid preservation solution at subzero temperatures^6–9^.

In this work, we employ a recently developed technique, isochoric supercooling^6^, to achieve high-stability and predictable supercooling of the University of Wisconsin (UW) organ preservation solution at −3 °C, in which we successfully preserve a functional 3D hiPSC-derived cardiac microphysiological system (MPS)^11^ for multiple days without the addition of non-physiological cryoprotectants such as dimethyl sulfoxide (DMSO) or glycerol.

## Results

### Cardiac MPS supercooled preservation protocol

**Fig. 1:**
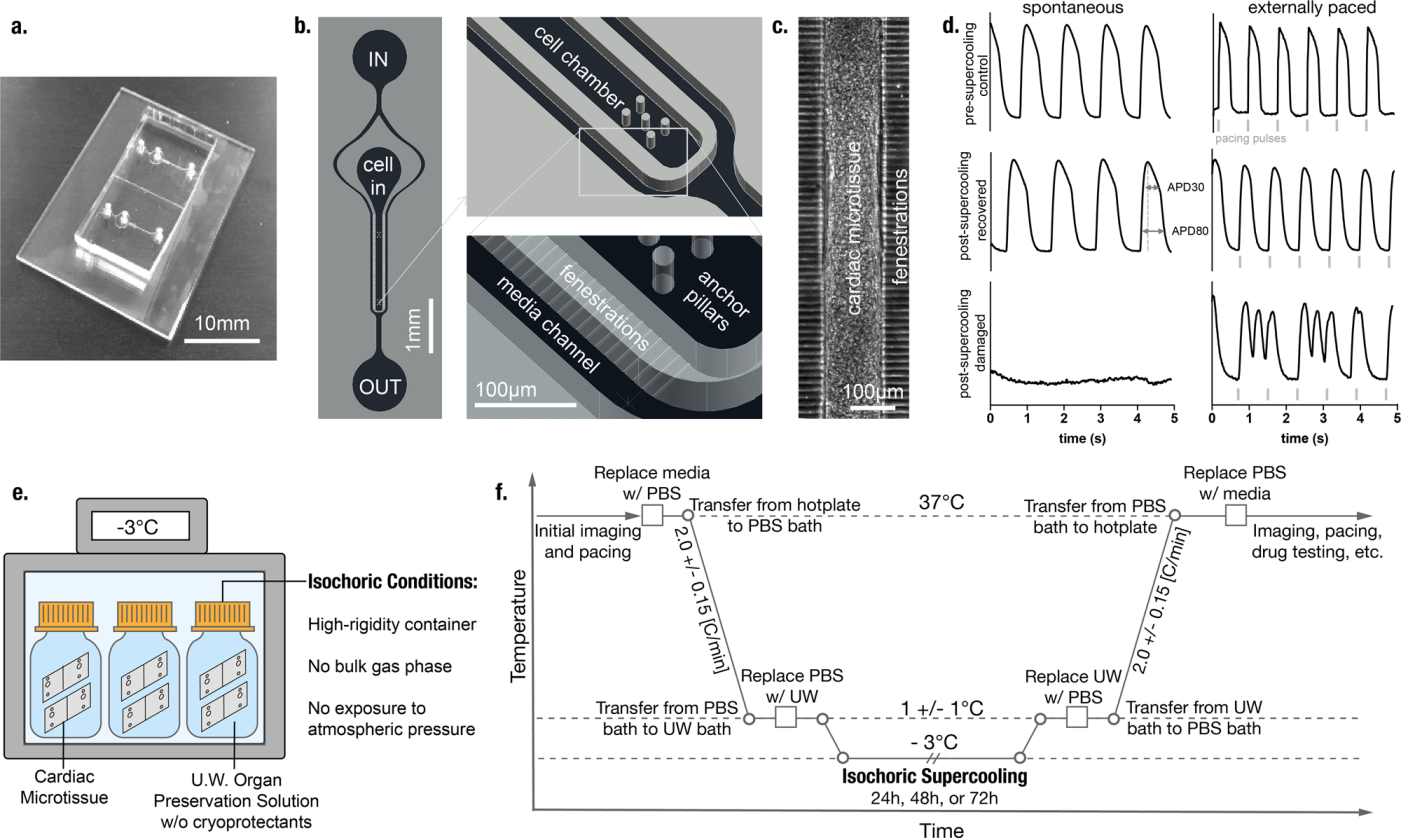
Cardiac microphysiological system and isochoric supercooling protocol. **a** A glass-PDMS microfluidic device featuring two independent MPS. **b** Details of the microfluidic features including media inlet (IN) and outlet (OUT) as well as the cell loading port (cell in), cell chamber with anchor pillars, media channels, and connecting fenestration channels. **c** Cardiac microtissue after cellular self-assembly within the cell chamber. **d** Example fluorescence traces of a genetically encoded calcium channel reporter (GCaMP) showing spontaneous and externally triggered (1.25 Hz) beating activity before cryopreservation as well as disturbed and restored tissue function after cryopreservation. The width of the calcium transient at 30% and 80% peak amplitude was measured as a proxy for action potential duration (APD30 and APD80, respectively). **e** Isochoric supercooling enables the preservation of biological matter in a metastable ice-free condition at temperatures below the freezing point of water/physiological saline. Isochoric conditions are achieved by confining the preservation solution in a high-rigidity container totally absent of bulk gas phase and denying it access to the atmospheric pressure reservoir, which alters both equilibrium thermodynamics and ice nucleation kinetics of the system^30,31^. **f** Temperature–time schematic of the isochoric supercooling preservation protocol.

The cardiac MPS combines human cells with microfluidics (Fig. 1a, b) to promote 3D self-assembly into a microtissue (Fig. 1c) that faithfully recapitulates complex human heart muscle structure and function^10–14^. The cardiac microtissues used in this work express a genetically encoded fluorescent calcium channel reporter^10^, a convenient means to optically and non-invasively track beating activity via calcium flux (Fig. 1d). The calcium transients can be analyzed to obtain important information on beating activity such as beat rate, beat duration (in this study we use calcium waveform as a proxy for action potential duration (APD)^11^), and aberrations in beating. The MPS also allows for external electrical tissue stimulation via pacing electrodes (Fig. 1d).

In designing the proof-of-concept preservation effort, we established the following criteria as indicators of successful preservation: retention of sarcomere structural integrity; retention of physiological function including autonomous beating activity and responsiveness to external electrical pacing stimuli; and retention of responsiveness to a pharmacological inotrope (here the β-adrenergic agonist, isoproterenol). Not only were these metrics rapidly monitored in the MPS, they can also be easily adapted to assess the preservation success of native heart tissue or full organs in a clinical setting (e.g., ECG, QT interval).

Inspired by previous supercooled preservation efforts involving the liver^7,8^, we designed a temperature–time trajectory by which to shuttle the cardiac MPS into and out of supercooled preservation. This trajectory is shown in Fig. 1f, and includes the following essential steps: replacing all metabolizable media in the MPS with non-metabolizable saline solution (PBS, no calcium); submerging the MPS in a bath of PBS initially at 37 °C, which is then cooled to 1 ± 1 °C at a rate of ~2 °C/min; replacing the PBS in the MPS with UW organ preservation solution (pre-chilled to 1 ± 1 °C); placing the MPS in a rigid isochoric container filled with pre-chilled UW organ preservation solution; sealing the container tightly without trapping any bulk gas phase^6^ (Fig. 1e); and, finally submerging the isochoric chamber in a constant-temperature circulating cooling bath at −3 °C for the target preservation duration (24, 48, or 72 h). Per Fig. 1f, these steps are performed in reverse for rewarming after preservation. After the MPS are returned to 37 °C and their supply of metabolizable media is replenished, they are imaged once without pacing, then moved to a cell culture incubator and allowed a minimum of 24 h of recovery before further imaging, pacing, and application of isoproterenol. A more detailed accounting of the protocol is available in the Methods section.

### Cardiac MPS supercooled preservation outcomes

**Fig. 2:**
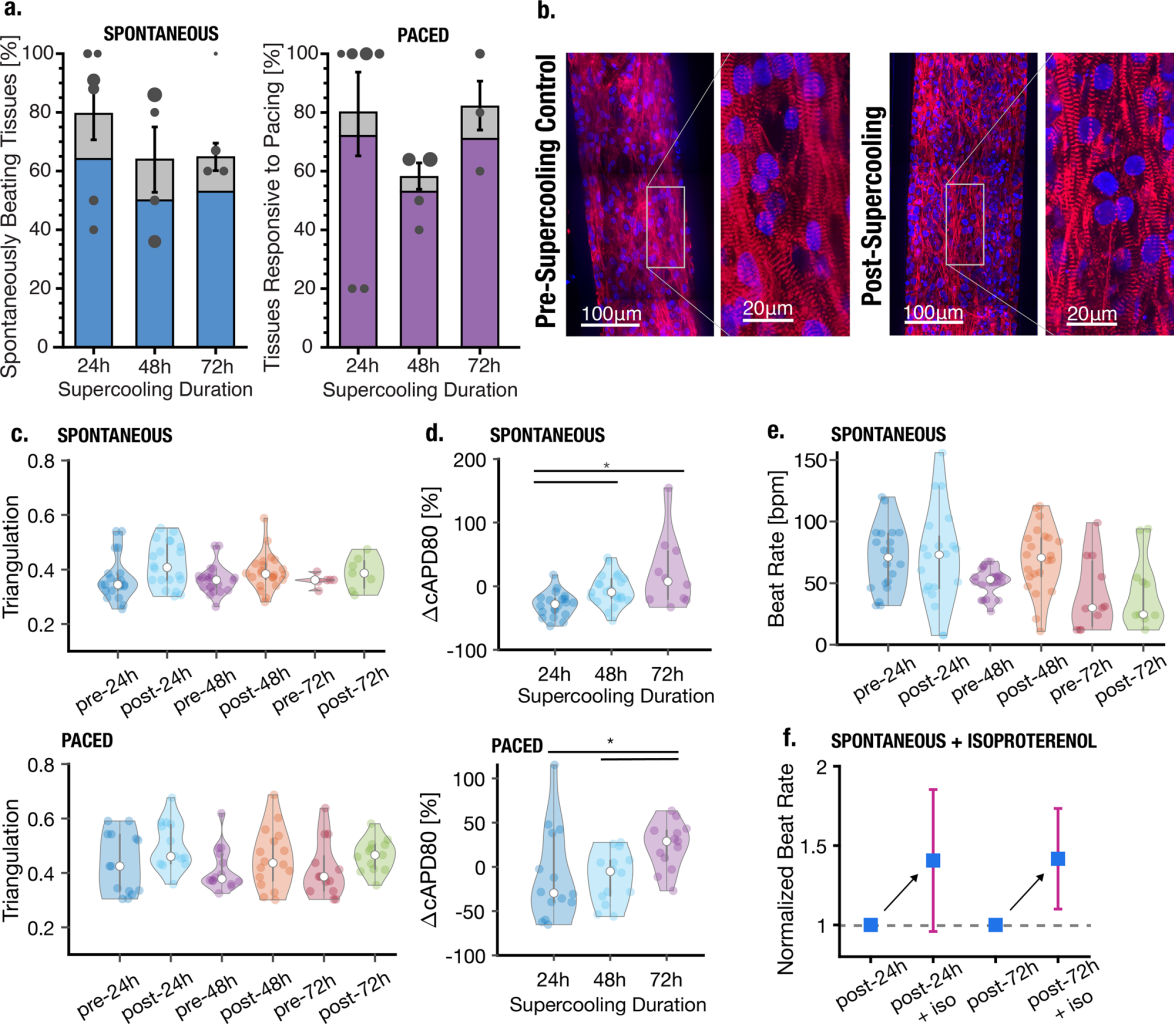
Recovery rate and comparison of key cardiac functionality parameters pre- and post- isochoric supercooling. Statistical difference between groups (*p* < 0.05) is marked by horizontal lines with asterisks. All raw data points are shown within violin plots. **a** Total recovery rate of preserved tissues as a function of preservation time. Gray bars indicate tissues that presented some activity post-isochoric supercooling, but were not coherent over the entire tissue (i.e., only part of the microtissue recovered). Scatter markers represent the weighted means of individual experiments, and marker diameter reflects the number of tissues (total sample sizes, i.e., number of independent cardiac tissues, for each condition: 24 h: *n* = 39; 48 h: *n* = 36; 72 h: *n* = 17). Error bars mark the standard error of the mean (SEM) across experimental groups, weighted by the total number of tissues evaluated in each group. These error bars reflect the SEM of the total surviving tissue population, i.e., the combined population of fully recovered and partially recovered tissues. **b** Confocal microscopy image of cardiac microtissue before and after 24 h supercooled preservation (α-actinin in red and nuclei in blue). **c** Triangulation ((APD80-APD30)/APD80) as a metric of beat shape following 24, 48, or 72 h isochoric supercooling. All raw data points are scatter plotted within the violin plots. **d** Percent change in beat rate-corrected APD80 of recovered tissues compared to pre-isochoric supercooling. **e** Spontaneous beat rate of recovered tissues. **f** Increase in normalized beat rate upon introduction of 1 μM isoproterenol for 30 min to tissues that had previously been subjected to 24 h (left) or 72 h (right) supercooled preservation (sample sizes: 24 h: *n* = 6; 72 h: *n* = 5).

The results of this preservation process are presented in Fig. 2. An average of 50–65% of tissues resumed normal spontaneous beating activity after isochoric supercooling (65% after 24-h preservation; 50% after 48-h preservation; 55% after 72-h preservation), and a similar percentage (50–70%) responded normally to external electrical stimulation (Fig. 2a), with no statistically significant differences observed between preservation durations. An additional 5–15% of MPS yielded the partial recovery of the tissue (gray bars in Fig. 2a), and the remaining 20–40% did not resume coherent beating. The explicit mechanistic drivers at play are not yet known and must be probed in future studies, but it should be noted that failure to resume coherent beating does not necessarily imply cell death—tissue beating is a combination of complex electrophysiological and mechanical processes reliant on myriad intra- and intercellular behavior, the corruption of any of which could contribute to failure.

Immunofluorescence staining confirmed qualitatively that the bulk structural integrity of the tissues was not altered by the cooling and warming processes, as shown by comparable post-preservation size and distribution of nuclei, as well as general alignment of cardiomyocytes and sarcomeric integrity (Fig. 2b and Supplementary Fig. S1). Further evaluation of physiological properties revealed that neither beat rate nor beat shape (as measured by a triangulation metric (APD80-APD30)/APD80^15^) was significantly altered by isochoric supercooling (Fig. 2c, e). Beat duration (APD80) showed a slight increasing trend with increasing supercooling both in spontaneous and externally paced conditions, though any potential biological relevance of this trend (Fig. 2d) is not apparent and should be explored in more detail in future studies.

In order to probe the conservation of drug response characteristics, we subjected the post-preservation MPS to 1 μM of the well-known β-adrenoreceptor agonist isoproterenol. The observed increase in spontaneous beat rate further confirmed the recovery of essential cardiac tissue function after isochoric supercooling, though the average beat rate increased by approximately 40% (Fig. 2f), which is less than previously reported for MPS created from the same hiPSC line^12^. It is unclear however whether this difference is a product of the supercooling process or a simple result of batch-to-batch variation within the microtissues, as the present tissues were not exposed to isoproterenol before supercooling (in order to avoid potentially confounding effects of pre-preservation drug exposure). The potential between supercooled preservation and altered drug response should be investigated further in future work.

With the exception of the slightly increasing trend in APD80, all recovery rates, electrophysiological parameters, and drug response characteristics did not vary significantly with supercooling duration (24, 48, or 72 h), suggesting that the mechanisms of tissue damage are not sharply dependent on metabolism and that significantly longer preservation periods may be achievable with similarly high recovery. We hypothesize that the majority of tissue damage occurs during the cooling/warming processes, which are independent of supercooling duration and were not fully optimized in this study. Excessive cooling rates may produce damaging stresses in the tissue due to a mismatch between the thermal contractility of the tissue and the surrounding microchamber. Damage may also be driven by the failure to sufficiently remove metabolizable or ionic constituents prior to cooling, which can generate toxicity during rewarming due to ionic imbalances or accumulation of metabolic byproducts^16^. It should also be noted that the observed failure of some MPS to resume beating may be a product of the inherent variability in the MPS themselves, which is evident in the pre-supercooling violin plots shown in Fig. 2c–e, and that this variability may mask smaller effects within the investigated parameter space.

Importantly, we observe no significant morphological or electrophysiological changes in recovered tissues that would suggest corruption of core cardiac functionality, as embodied most essentially by the resumption of spontaneous autonomous beating with an unchanged beat shape and expected responsiveness to isoproterenol treatment.

## Discussion

Our data demonstrate that functional 3D microtissues may be effectively preserved at subzero temperatures, which has several important implications.

First, these microtissue constructs may offer essential translatable insights into expected physiological effects of low-temperature preservation at the whole-organ scale. Indeed, in the drug response and pharmacokinetic analysis domains, various human-derived MPS have been demonstrated to be superior predictors of clinical response compared to animal models, offering closer physiologic and pharmacokinetic properties to native tissue whilst enabling higher-throughput testing^10,17–19^. Furthermore, recent work has demonstrated that the cardiac MPS can successfully predict clinical trial arrhythmia outcomes^11^, indicating potential prognostic value in predicting human-scale cardiac behavior. We suggest that these same advantages can apply to low-temperature preservation, and that MPS may provide a better testbed for identifying the missing links required for timely translation of benchtop preservation protocols to the clinic, where advances in organ preservation are urgently needed. Translatability to full-organ systems will of course require computational analysis of thermal/mass transport and metabolic scaling issues, as well as accounting for the fundamental differences in structural and electrophysiological complexity between the MPS and the native system. However, the complex functional outputs that can be probed with the MPS may move benchtop preservation research a significant step closer to clinical relevance.

Second, the high-recovery preservation results obtained using the protocol described herein, which does not include the introduction of non-physiological cryoprotectants, suggest that MPS platforms may also be used to study the explicit effects of various cryoprotectants on preservation efficacy. MPS platforms could be used to simultaneously screen cryoprotectants for toxicity (in a complex genetically human context), cryoprotective action, and diffusion/perfusion performance. The use of non-physiological cryoprotectants has been omnipresent in cellular preservation protocols since the 1950s, but has failed to penetrate clinical full-organ preservation, largely due to concerns surrounding toxicity or perfusion in native human tissue, and poor scaling of cell preservation results^5,20^. Our data establish a baseline from which MPS may be used for higher-throughput, higher-reliability cryoprotectant screening.

This work also, to the best of our knowledge, presents the first demonstration of biological preservation by isochoric supercooling^6^. This technique provides a thermodynamically simple and procedurally streamlined method of ice-free preservation at sub-0 °C temperatures, and given its non-reliance on specific cryoprotective agents, we suggest that it may be applied for the preservation of arbitrary biologics in the high-subzero temperature range.

Building upon the baseline data, future work may probe a wide variety of additional complex functional parameters available from the cardiac MPS. Perhaps most pressingly, the contractile force and relaxation should be analyzed post-supercooling, which can provide a more definitive measure of health and function in the revived tissues. Additional analyses may include metabolic activity, biomarker secretion, or PCR studies.

In summary, this study demonstrates successful isochoric supercooled preservation and recovery of microfluidic human cardiac tissue cultures based on structural and functional characteristics. Further molecular biological studies will be needed to assess any changes in gene and biomarker protein expression levels, and optimization of thermal cycling protocols should be explored to maximize recovery rate. Moving forward, as MPS platforms continue to advance in both fidelity and accessibility^21,22^, we suggest that MPS preservation studies may provide a useful preliminary screening tool for any full-organ or complex tissue preservation efforts, providing insight into potential structural and functional effects of the preservation process on high-content, complex and human-derived tissues.

## Methods

### Preservation—cooling

The cooling protocol follows the trajectory plotted in Fig. 1f. First, on a 37 °C hotplate (Tokai Hit, Gendoji-cho, Japan), each MPS was flushed by gravity perfusion with 200 μL phosphate-buffered saline (no calcium) in order to remove metabolizable compounds from the MPS prior to cooling. This step was performed to minimize the generation of metabolic byproducts during cooling that may accumulate during storage and cause toxicity upon return to normothermia. Importantly, PBS without calcium was employed during cooling and warming in order to minimize the risk of calcium shock or other ionic imbalance effects as tissues warm-up and get reperfused with culture media. The surface of the MPS device was briefly wiped with 70% ethanol to reduce contamination risks. The MPS were then submerged in a well of ~30 mL of PBS at 37 °C. This well was then placed directly into a circulating ice and water bath at 1 ± 1 °C, resulting in a cooling rate of 2.1 ± 0.15 °C/min. The temperature in both the well and a reference MPS were continuously monitored via a thermocouple. When the reference MPS temperature reached below 2 °C, all MPS were submerged in an identical well filled with equally cold University of Wisconsin (UW) organ preservation solution. The MPS were then flushed with 200 μL of pre-chilled UW solution by gravity perfusion.

### Preservation—storage under isochoric supercooling

Following cooling, the MPS were transferred to an approximately 65 mL thick-walled glass container filled with pre-chilled UW solution at 1 ± 1 °C. Crucially, care was taken to ensure that no bulk gas phase (e.g., air bubbles) were present in the solution before and after the introduction of the MPS. The container was filled to the brim and then sealed using a rigid threaded polypropylene cap that included a centered plug to displace air and liquid as the cap is threaded down, and ensures that the sealed container contains no air. A more detailed accounting of this assembly process, the described components, and the thermodynamic and kinetic effects of confining the preservation liquid in constant-volume (isochoric) conditions absent air are available in our previous publication^23^.

The isochoric container, housing 2–8 MPS per container, was then submerged in a constant-temperature circulating cooling bath (PolyScience, USA) at −3 °C for 24, 48, or 72 h. This preservation temperature was chosen based on our previous isochoric nucleation experiments showing that isochoric supercooling was extremely stable (e.g., the likelihood of ice nucleation was <1%) at −3 °C for physiological saline, which has a near-identical freezing point to UW solution (−0.56 °C). The stability of isochoric supercooling is a multifaceted consequence of denying the system access to a pressure reservoir (e.g., the atmosphere), which suppresses density fluctuations^23^; denying the system access to air–water interfaces, which may function as nucleation sites^23,24^; and suppressing secondary nucleation mechanisms such as cavitation-induced nucleation^25^.

### Preservation—warming

Per Fig. 1f, in order to warm the MPS back to physiological temperatures, the cooling protocol was applied precisely in reverse. It should be noted in particular that the isochoric container was warmed from −3 °C back to 1 ± 1 °C (via ice bath) before it was opened. Premature opening of the isochoric container (and disruption of isochoric thermodynamic conditions) at temperatures less than −0.56 °C can lead to immediate ice formation.

### Recovery

In initial protocols, the MPS were imaged for spontaneous activity on a hotplate directly the following warming and before being placed back into an incubator at 37 °C with (5% CO_2_). Of note, in this directly post-warming measurement, incoherent electrophysiological activity was visually observed in several MPS. Constellations of calcium transients could be observed in various parts of the tissue, even while coherent signaling across the whole tissue (as accompanies autonomous beating) was absent. Early in our study design, this observation indicated to us that tissues may not be dead, even while not spontaneously beating, and we thus elected to employ a minimum-24-h recovery period before electrical pacing and further imaging. Later experiments had an adjusted protocol where MPS were directly put in the incubator after warming (skipping the previously described imaging), to rapidly expose the tissues to physiological temperature and CO_2_. The results reported in Fig. 2, all reflect incubated recovery times of at least 24 h (minimum 24 h, maximum 96 h). We did not distinguish between recovery times in aggregating the data presented in Fig. 2, as statistical analysis of the recovery rate based on supercooling duration and recovery time showed no significant difference between recovery times >24 h. However, while outside of the scope of this study, the dedicated high-time granularity study of the recovery process within the first 24 h post-supercooling may yield important insight into the physiological effects of thermal cycling and prolonged hypothermic storage, and the cardiac recovery process. Similarly, continued observation beyond 96 h post-preservation will give insight into the long-term survival and stability of previously supercooled MPS.

### Microfluidic device design and fabrication

Cardiac MPS were designed and prepared as previously described^10,12,26^. In brief, the MPS were fabricated using traditional soft lithography. Polydimethylsiloxane (PDMS; Dow Chemical, Sylgard 184) was used for the fabrication of our MPS because of its well-established minimal response to large temperature variations, aging, oxidation, moisture, or ultraviolet radiation. It does not show significant changes in viscosity, oxygen permeability, or mechanical properties upon temperature changes in a range from −40 to >150 °C. Moreover, the high flexibility of PDMS allows it to be compressed, stretched, and bent repeatedly^27^.

A 10:1 mixture of PDMS with curing agent was prepared, mixed thoroughly, and poured onto silicon wafers that served as master molds featuring the negatives of the microfluidic structures made from SU-8. After curing, the PDMS was peeled off the master mold and cut to size. Holes of 0.75 mm diameter were punched out for access ports. Oxygen plasma surface treatment (21 W, 24 s, 600 mTorr) was applied to permanently bond the PDMS to 1 mm glass slides (VWR, 48300-048) cleaved to 2.5 × 1.5 cm (Fig. 1a).

The microfluidic design featured elongated cell chambers that promote cellular self-assembly into a uniaxially beating microtissue (Fig. 1b). The chamber dimensions varied from 800 to 1800 µm length; 120 to 170 µm width and 60 to 70 µm height. Media channels ran in parallel on either side of the cell chamber. Media exchange in the cell chamber was achieved via an array of small connection channels (2 × 2 µm cross-section; 40 µm length) that protected the tissue from fluid mechanical forces^10^. Small pillars (20 µm diameter) at either end of the cell chamber served as structural anchor points to keep the muscle fiber from collapsing. Each microfluidic device featured either a single tissue chamber or 4 identical tissue chambers in parallel.

### CM differentiation from iPSC

Cell culture was performed similarly to already published protocols^12^. We used the human iPSC line Wild Type C (WTC; # GM25256, Coriell Institute) edited to express a fluorescent reporter for intracellular calcium (GCaMP6f)^28^. After thawing the iPSCs were cultured on Matrigel (Corning, 354277) in mTeSR-1 media (Stem Cell Technologies, 85851) for several passages every 3 days before differentiation was started. Accutase (Millipore, SCR005) was used for dissociation. Differentiation was induced via the Wnt/β-catenin signaling pathway. First, 8 µM CHIR 99021 (Peprotech, 2520691-10MG) was applied for 24 h in RPMI 1640 media (Gibco, 11875-093) with 2% of B27 Minus Insulin (B27-I, Gibco, A18956-01), followed by 24 h in RPMI + B27-I without any small molecules. Then, 5 µM IWP-4 (Peprotech, 6861787-10MG) was applied for 48 h, followed by 48 h in RPMI + B27-I. CM differentiation was completed with subsequent 48 h media exchanges with RPMI + 2% B27 supplement that contained insulin (B27 + C, Gibco, 17504-044) until the cells began to beat spontaneously. CM were used up to 14 days after differentiation. All studies involving human iPSCs were reviewed and approved by the UC Berkeley Stem Cell Oversight Committee.

### Cardiac MPS loading and maintenance

For MPS loading, cells were singularized using collagenase type 2 (Worthington Biochemical Corporation, LS004176) for 45–60 min in a cell culture incubator and collected in EB20 media (Knock Out DMEM (Gibco, 10829-018) with 20% FBS (Gibco, 16000-044), 1% MEM non-essential amino acids (Gibco, 11140-050), 1% Glutamax (Gibco, 35050-061) and 400 nM 2-Mercaptoethanol (Gibco, 21985-023)) supplemented with 10 µM ROCK-inhibitor (RI, Y-27632 dihydrochloride, Peprotech, 1293823-10MG). Each cell chamber was loaded with 3 µL cell suspension containing enough cells to completely fill the chamber (between 5000 and 20000 cells). The cell suspension was injected into the cell loading port using a pipette tip (Rainin, LTS ultra fine 20 μL), which was cut to about 1 cm in length and remained in the chip. Two centrifugation steps (3 min at 300 rcf; first horizontal than vertical orientation of the chip) were performed to aggregate the cells and move them into the cell chamber. The cell loading port was then sealed and EB20 + RI media was perfused through the media channels after 1 h incubation time. The next day media was exchanged to RPMI + 2% B27 + C and changed three times per week thereafter. MPS were used within 30 days after loading.

### Data acquisition

Videos were obtained using a digital CMOS camera (HAMAMATSU, C11440/ORCA-Flash 4.0) mounted to Nikon TE-300 inverted microscope with an LED light engine (Lumencor SpectraX). A heated platform (Tokai Hit, TPi-SQX) was used to image the MPS at 37 °C. For electrical stimulation, a pulse generator (ION OPTIX Myopacer Field Simulator) was used. 20 V biphasic rectangular pulses (20 ms) were applied via 1.5″ long blunt stainless-steel needles (Vita Needle M937) inserted into the media inlet and outlet pipette tips. Calcium traces were recorded at 100 fps with 4 × 4 binning using the cyan LED for excitation. A 1 mM isoproterenol stock was prepared freshly from isoproterenol hydrochloride (I0260; TCI Chemicals) powder right before the drug response experiments, sterile filtered and diluted to 1 µM. 100 µL of drug-containing media were applied to each chip via the inlet pipette tip 30 min before recording.

### Data analysis

Fluorescence videos were converted into time-intensity profiles (for example Fig. 1d) using an in-house library of Python scripts. Beat parameters such as beat rate and peak duration at 30% and 80% intensity (APD30 and APD80, respectively) were automatically detected. Further metrics were calculated: The beat rate-corrected peak duration (cAPD80) was computed using the Fridericia method, which corrects for variations in beat rate by scaling the values to 1 Hz beat rate^29^: ${{{{{{{\mathrm{cAPD}}}}}}}}_{80}={{{{{{{\mathrm{APD}}}}}}}}_{80}/{{{{{{{\mathrm{BR}}}}}}}}^{-\frac{1}{3}}$ with BR being the beat rate in Hz and cAPD_80_ being the peak duration at 80% peak height corrected to a beat rate of 1 Hz. Triangulation, as introduced by Hondeghem et al.^15^, was computed as a metric of beat shape: $\left({{{{{{{\mathrm{APD}}}}}}}}_{80}-{{{{{{{\mathrm{APD}}}}}}}}_{30}\right)/{{{{{{{\mathrm{APD}}}}}}}}_{80}$. Lower triangulation values indicate a more rectangular beat shape while higher values indicate stronger triangulation, which is associated with increased pro-arrhythmic potential.

For analysis of the recovery rate, we counted the percentage of tissues that resumed beating activity after preservation as judged by contractile tissue motion or peaks in the calcium trace. We distinguished between samples that showed coherent beating of the entire tissue as one unit from those that showed activity only in a portion of the tissue. We defined recovery rate as the number of tissues that resumed beating activity spontaneously or in response to electrical stimulation at any time point after preservation, divided by the total number of preserved tissues.

### Statistics and reproducibility

All graphs were plotted in MATLAB (version R2019b). Violin plots were generated using a third-party MATLAB script developed by Bastian Bechtold (available here: https://github.com/bastibe/Violinplot-Matlab). Figures were assembled using Adobe Illustrator (version 25.0.1). Statistical analysis to compare preservation effects was done by one-way ANOVA, with Bonferroni’s method used for multiple comparisons test (all performed in MATLAB using built-in functionality). All samples evaluated herein are biologically independent, i.e., no technical repeats on the same tissue were performed. Post-preservation results were compared to pre-preservation results for each condition, as well as to other post-preservation results at different supercooling timepoints. Effects were reported for significance levels of *p* < 0.05 (*). In the presented data, horizontal bars topped by an asterisk indicate a significant difference between groups, and if no bars are shown, the groups were determined to be statistically similar.

### Immunofluorescence

After preservation, the MPS were allowed to warm up and resume beating. After 24 h of recovery, tissues were washed with phosphate-buffered saline (PBS) for 5 min and then perfused with 4% paraformaldehyde (PFA) for 15 min. After another two washing steps with PBS, the PDMS was carefully filleted off the glass slides using a scalpel to expose the tissues. The tissues, which remained attached to the PDMS, were sequentially submerged in the following solutions: blocking buffer (BB: 1% BSA 10% FBS 0.5% Triton 0.05% sodium azide) overnight at 4 °C; DAPI 1:1000 (Invitrogen D1306) in BB for 30–40 min at 25 °C; primary antibody (Mouse anti-α-actinin, Life technologies 41811) 1:100 in BB for 48 h at 4 °C; two washing steps in BB for 2 h at 25 °C and 1 washing step overnight at 4 °C; secondary antibody (Goat anti-mouse IgG Alexa 568 H + L, Life Technology a11004) 1:100 in BB overnight at 4 °C; two washing steps in BB for 2 h at 25 °C and 1 washing step overnight at 4 °C. All incubation steps were performed on a shaker. Tissues were imaged in the Opera Phenix™ High Content Screening System using a Proprietary Synchrony™ Optics 63x water immersion lens and Harmony acquisition software. We performed z-stacks over 60μm height with a step size of 0.5μm. Image processing in ImageJ was done to stitch large images, perform maximum intensity projection (15 slices) and enhance contrast.

### Reporting summary

Further information on research design is available in the Nature Research Reporting Summary linked to this article.

## Supplementary information

Supplementary Information

Description of Supplementary Files

Supplementary Data 1

Reporting Summary

## Data Availability

All data are available upon reasonable request. All data points are plotted in Figs. 1 and 2 are available in the Supplementary Data 1 file.
