# Supplementary material for: Isochoric supercooled preservation and revival of human cardiac microtissues
Source: Commun Biol. 2021 Sep 22;4:1118. doi: 10.1038/s42003-021-02650-9 (PMC8458396; doi:10.1038/s42003-021-02650-9)
Supplement: Supplementary file 1 — Supplementary Information [file 42003_2021_2650_MOESM1_ESM.pdf]

Isochoric supercooled preservation and revival of human cardiac microtissues

Supplementary Information

Supplementary Figures

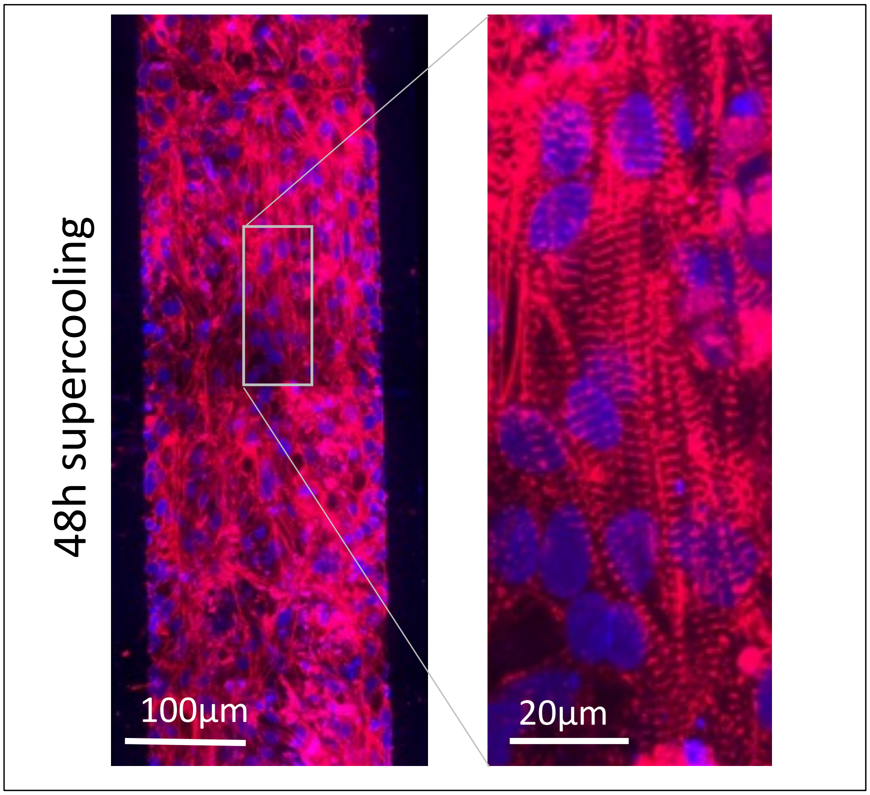

Figure S1. Confocal microscopy image of cardiac microtissue after 48h supercooled preservation (α-actinin in red and nuclei in blue), showing clear sarcomere definition, integrity, and alignment.
