# Supplementary material for: Isochoric supercooled preservation and revival of human cardiac microtissues
Source: Commun Biol. 2021 Sep 22;4:1118. doi: 10.1038/s42003-021-02650-9 (PMC8458396; doi:10.1038/s42003-021-02650-9)
Supplement: Supplementary file 2 — Description of Supplementary Files [file 42003_2021_2650_MOESM2_ESM.pdf]

Description of Additional Supplementary Files

File name: Supplementary Data 1

Description: Collection of all data points plotted within the main text figures.
